# Association Between Physical Therapy and Mortality in Older Patients with Heart Failure and Chronic Kidney Disease

**DOI:** 10.3390/jcm15187043

**Published:** 2026-09-11

**Authors:** Toshimi Sato, Tomoyuki Morisawa, Masakazu Saitoh, Kentaro Iwata, Michitaka Kato, Koji Sakurada, Yuji Kono, Daisuke Suzuki, Tetsuya Takahashi

**Affiliations:** 1 Department of Physical Therapy, School of Health Sciences, Fukushima Medical University, Fukushima 960-8516, Japan; 2 Committee of the J-Proof HF Registry, Japanese Society of Cardiovascular Physical Therapy, Tokyo 106-0032, Japan; t.morisawa@kzc.jp (T.M.); m.saito.tl@juntendo.ac.jp (M.S.); iwaken@kcho.jp (K.I.); katomanzooo@gmail.com (M.K.); sakura282517@gmail.com (K.S.); yt-kono@fujita-hu.ac.jp (Y.K.); mh258517@fmu.ac.jp (D.S.); te-takahashi@juntendo.ac.jp (T.T.); 3 Department of Rehabilitation, Kobe Rehabilitation Hospital, Kobe 651-1106, Japan; 4 Department of Physical Therapy, Faculty of Health Science, Juntendo University, Tokyo 113-8421, Japan; 5 Department of Rehabilitation, Kobe City Medical Center General Hospital, Kobe 650-0047, Japan; 6 Department of Health Sciences, Major in Rehabilitation Science, Nagoya City University, Nagoya 467-8601, Japan; 7 Department of Rehabilitation, Sanai Hospital, Saitama 338-0837, Japan; 8 Department of Rehabilitation, Fujita Health University Hospital, Toyoake 470-1192, Japan; 9 Department of Rehabilitation, Southern Tohoku General Hospital, Koriyama 963-8563, Japan

**Keywords:** heart failure, chronic kidney disease, physical therapy, cardiac rehabilitation, mortality, older adults

## Abstract

**Background/Objectives**: Evidence regarding the prognostic impact of outpatient physical therapy (PT) initiated shortly after discharge in older patients with chronic kidney disease (CKD) hospitalized for worsening heart failure (HF) is limited. We evaluated the association between outpatient PT initiation within 30 days of discharge and the primary endpoint of all-cause mortality within 1 year after discharge. **Methods**: In this retrospective multicenter cohort study, we applied a 30-day post-discharge landmark design. Patients who died or were rehospitalized within 30 days after discharge were excluded, and 6359 eligible 30-day event-free survivors were included in the analysis. Of these, 244 (3.8%) underwent outpatient PT within 30 days of discharge, and the remaining patients had no documented outpatient PT within that period. Propensity scores were estimated from prespecified covariates and used for 1:1 matching. We assessed associations with 1-year all-cause mortality, cardiovascular (CV)-related rehospitalization, and their composite using Cox proportional hazards models in the propensity score-matched cohorts and pooled estimates across the 50 imputed and matched datasets using Rubin’s rules. **Results**: Within 1 year of discharge, 1099 participants died, 1586 were rehospitalized for CV-related reasons, and 2325 experienced either event. In pooled Cox regression analyses, outpatient PT initiation within 30 days was associated with lower all-cause mortality (hazard ratio [HR], 0.46; 95% confidence interval [CI], 0.22–0.97; *p* = 0.042) and the composite outcome (HR, 0.67; 95% CI, 0.48–0.94; *p* = 0.021), but not CV-related rehospitalization alone (HR, 0.78; 95% CI, 0.55–1.10; *p* = 0.178). **Conclusions**: Among patients with HF and study-defined CKD who were alive and free from CV rehospitalization at the 30-day landmark, early outpatient PT participation was associated with a lower risk of subsequent all-cause mortality. Given the observational design, treatment selection, and residual confounding, this finding does not establish a causal effect and requires confirmation in prospective controlled studies.

## 1. Introduction

Heart failure (HF) is associated with high morbidity and mortality rates among older adults, in whom disease pathophysiology is frequently complicated by age-related multi-organ dysfunction. Japan is the most rapidly aging society worldwide, with 29.3% of its population aged ≥65 years in 2024 [1]. This demographic transition has led to a substantial increase in the prevalence of HF, resulting in a growing burden on healthcare systems through recurrent hospitalizations, functional decline, and excess mortality [2,3,4]. These challenges underscore the importance of effective post-discharge management strategies aimed at improving the long-term prognosis of older patients with HF.

Kidney dysfunction is highly prevalent among such patients; more than 40% reportedly have concomitant chronic kidney disease (CKD) [5,6]. Age-related declines in kidney functional reserve, progressive vascular changes, and vulnerability to kidney hypoperfusion in the setting of polypharmacy contribute to this high comorbidity rate [7,8,9]. Importantly, CKD is an established independent predictor of adverse outcomes in HF, including an increased mortality risk, higher rehospitalization rate, and impaired health-related quality of life [5,6,10,11]. Consequently, the identification of modifiable post-discharge interventions that may improve survival and reduce cardiovascular (CV)-related rehospitalization, including hospitalization for HF, remains a critical clinical challenge in this high-risk population.

Exercise-based cardiac rehabilitation (CR) is a cornerstone of guideline-directed management for HF [12,13,14,15]. In clinical practice in Japan, structured exercise-based CR after hospital discharge is predominantly delivered as outpatient physical therapy (PT). PT entails exercises based on patients’ physical function, comorbidities, and living environments, with the goal of optimizing their physical and functional capacity during the vulnerable post-discharge period. Such interventions reportedly contribute to improved physical functioning [16] and long-term survival rates [17] in patients with HF who are also frail.

However, evidence on the association between post-discharge PT and survival outcomes remains limited, particularly among older patients with both HF and CKD. Moreover, observational studies of post-discharge rehabilitation are susceptible to time-related biases if exposure definitions are not rigorously specified. Therefore, high-quality real-world analyses in which immortal time bias is explicitly addressed and which are robustly controlled for confounding factors are needed to clarify the prognostic significance of PT in older patients with comorbid HF and CKD. Accordingly, this study investigated whether outpatient PT participation within 30 days after discharge was associated with the primary endpoint of all-cause mortality within 1 year after discharge among patients with HF and study-defined CKD who were alive and free from CV rehospitalization at the 30-day landmark.

## 2. Materials and Methods

### 2.1. Study Design and Patients

This was a retrospective multicenter cohort study using prospectively collected data from the J-Proof HF registry [18], which enrolled patients hospitalized for acute HF at 96 institutions in Japan. From December 2020 to March 2022, consecutive patients aged ≥65 years who were prescribed PT during hospitalization for worsening HF were enrolled. All patients received acute-phase treatment and PT during hospitalization.

Patients were excluded if they died in the hospital; were discharged within 3 days; lacked data regarding the baseline estimated glomerular filtration rate (eGFR), follow-up outcome, or PT participation during the follow-up period; had an eGFR ≥60 mL/min/1.73 m^2^; or were on maintenance dialysis.

Details of the J-Proof HF registry are available in the University Hospital Medical Information Network (UMIN) Clinical Trials Registry (ID: UMIN000047893). The study protocol was approved by the Ethics Committee of Juntendo University School of Health Sciences, Tokyo, Japan (reference no. 19-005), as well as the ethics committees of all 96 participating institutions. Written informed consent or opt-out consent was obtained from all participants, according to the regulations of each institution’s ethics committee. The study complied with the principles of the Declaration of Helsinki. Additionally, the protocol for this secondary analysis of anonymized data obtained from the registry was approved by the Ethics Committee of Fukushima Medical University (reference no. REC2025-156).

### 2.2. Demographic and Clinical Data

#### 2.2.1. Demographic and Clinical Characteristics

Demographic and clinical characteristics included age, sex, primary diagnosis, comorbidities, smoking status, length of hospital stay, history of HF hospitalization, and living arrangement (whether the patient lived alone). For this study, CKD was operationally defined as an eGFR <60 mL/min/1.73 m^2^ during the index hospitalization. Longitudinal eGFR measurements sufficient to establish persistence for at least 3 months and albuminuria data were unavailable; therefore, this study-specific definition did not establish CKD according to formal diagnostic criteria for every patient.

#### 2.2.2. Laboratory and Echocardiographic Variables

Laboratory and echocardiographic variables included left ventricular ejection fraction, left atrial diameter, E/e′, BNP or NT-proBNP, serum creatinine, serum albumin, hemoglobin, sodium, and eGFR. Because natriuretic peptide assays varied across centers, NT-proBNP values were converted to BNP equivalents using the validated formula reported by Ishihara et al. [19].

#### 2.2.3. Physical Function and Frailty

Discharge physical function measures, including grip strength, comfortable walking speed, and the Barthel Index, were collected. Frailty was assessed using the Kihon Checklist (KCL), a 25-item self-reported questionnaire developed by the Japanese Ministry of Health, Labour and Welfare to screen for the risk of functional decline in older adults. Activities of daily living (ADL) dependency was defined as a Barthel Index ≤60 at discharge. As in a previous study, a KCL score ≥8 was defined as frailty [20]. The KCL was assessed through patient or family interviews.

#### 2.2.4. Discharge Medications

Information on discharge medications was collected for all participants.

### 2.3. PT

Early outpatient PT participation was defined pragmatically as at least one documented outpatient PT session at a participating institution within 30 days after discharge. Patient-level information on the PT initiation date and cumulative number of documented sessions was available. However, individual session dates, daily treatment status, treatment intensity, and reliable treatment-discontinuation dates were unavailable. For descriptive purposes, the cumulative number of sessions was categorized as 1–4, 5–9, or ≥10 sessions among PT participants. The primary exposure therefore represented early participation rather than completion of a standardized program or a defined physiological treatment dose.

At participating institutions, outpatient PT was generally delivered within the framework of Japanese Circulation Society cardiac rehabilitation guidelines [21]. These guidelines describe progression from supervised exercise to a combination of supervised and home-based exercise. However, the registry did not verify whether each participant received the guideline-recommended frequency or duration; therefore, the observed exposure should not be interpreted as completion of a 5-month program.

Exercise was individualized according to baseline function and rehabilitation goals, starting with stretching and moderate-intensity aerobic exercise, with resistance training introduced once the patient’s clinical condition had stabilized.

Aerobic exercise intensity was generally prescribed at the anaerobic threshold level, guided either by the target heart rate (determined using the Karvonen formula [*k* = 0.4–0.6]) or by a perceived exertion of 12–13 on the Borg scale. In addition to exercise therapy, patients and caregivers received guideline-based education on HF management. Participation in PT was documented by site investigators based on data extracted from the electronic medical records.

The non-PT group comprised patients with no documented outpatient PT session at a participating institution within 30 days after discharge. The registry did not systematically capture information on home exercise instructions, community rehabilitation, nursing rehabilitation, or PT delivered outside participating institutions.

### 2.4. Outcomes

The primary outcome was all-cause mortality within 1 year of discharge. Secondary outcomes included CV rehospitalization and the composite of all-cause mortality and CV rehospitalization.

CV rehospitalization was defined as the first rehospitalization for HF or for any other CV-related cause within 1 year of discharge. Detailed diagnostic subcategories other than HF were not available in the registry. The composite outcome was defined as the first occurrence of all-cause death or CV-related rehospitalization within 1 year of discharge. Information on rehospitalization events was obtained through postal surveys, telephonic follow-up, and review of the electronic medical records. Rehospitalization events were reported by participating sites and were not centrally adjudicated by an independent clinical events committee.

### 2.5. Statistical Analysis

To mitigate immortal time bias, we implemented a prespecified 30-day landmark design by resetting time zero (t0) to 30 days after hospital discharge. We ensured that treatment assignment and the start of follow-up were aligned to preserve the prespecified treatment-strategy definitions. Hence, we excluded patients who died or were rehospitalized within 30 days of discharge or who initiated outpatient PT after 30 days. Follow-up for the primary landmark analyses began at the landmark time (t0 = day 30) and continued until the first occurrence of the outcome of interest or administrative censoring at 1 year after discharge (i.e., 335 days after t0), whichever came first.

Kidney function was categorized according to Kidney Disease: Improving Global Outcomes criteria as CKD stage 3a (eGFR, 45.0–59.9 mL/min/1.73 m^2^), 3b (30.0–44.9 mL/min/1.73 m^2^), and 4–5 (<30.0 mL/min/1.73 m^2^) [22]. Baseline characteristics were summarized as means (standard deviations) for continuous variables and frequencies (percentages) for categorical variables. They were compared using unpaired *t*-tests for continuous variables and chi-square or Fisher’s exact tests for categorical variables, as appropriate.

Missing baseline covariates were imputed using multiple imputation by chained equations under the missing-at-random assumption, generating 50 imputed datasets with the mice package version 3.17.0 in R software version 4.4.1 (R Foundation for Statistical Computing, Vienna, Austria) [23]. All variables included in subsequent analyses were entered into the imputation models. BNP values were log-transformed (after adding a constant of 1 × 10^−6^) to address skewness, and the log-BNP variable was used in both imputation and subsequent models to satisfy linearity assumptions [19]. Variable-specific missingness is summarized in Online Table S1.

Propensity scores for early outpatient PT participation were estimated using a multivariable logistic regression model that included prespecified baseline covariates: demographic characteristics (age, sex, and body mass index); clinical and laboratory measures (serum albumin, hemoglobin, and log-transformed BNP levels); comorbidities (diabetes mellitus, hypertension, dyslipidemia, chronic obstructive pulmonary disease, musculoskeletal disease, cerebrovascular disease, cancer, and dementia); HF-related characteristics (history of HF, NYHA functional class, HF with reduced ejection fraction, and history of ischemic heart disease); kidney-function category; frailty status; functional status (Barthel Index and comfortable walking speed); social and lifestyle factors (living alone and current smoking); and medications at discharge (angiotensin-converting enzyme inhibitors, angiotensin II receptor blockers, or angiotensin receptor-neprilysin inhibitors; β-blockers; diuretics; and sodium-glucose cotransporter 2 inhibitors). Propensity score matching (PSM) was performed separately within each of the 50 imputed datasets using 1:1 nearest-neighbor matching without replacement, implemented with the MatchIt and MatchThem packages in R. The propensity score estimated from the logistic regression model was used as the matching distance, and no caliper restriction was applied. The matching analysis targeted the average treatment effect among the treated participants. Propensity-score overlap was assessed by examining the distributions and empirical ranges of the estimated propensity scores in the PT and non-PT groups within each imputed dataset. Covariate balance before and after matching was evaluated using absolute standardized mean differences, with values <0.10 indicating acceptable balance, and visualized using Love plots. Balance statistics were summarized across the 50 imputed and matched datasets.

In the PSM cohorts, we fitted unadjusted Cox proportional-hazards models with outpatient PT as the exposure. No additional covariate adjustment was performed because matching achieved acceptable balance across the prespecified baseline covariates. The limited number of mortality events precluded reliable multivariable adjustment without a substantial risk of overfitting and model instability. CV rehospitalization was also analyzed using Fine–Gray subdistribution hazard models, treating death as a competing event. Cox and Fine–Gray model coefficients and standard errors were estimated separately in each of the 50 imputed and matched datasets and pooled using Rubin’s rules [23].

As a sensitivity analysis, we fitted a Cox proportional-hazards model in which outpatient PT initiation was treated as a time-dependent exposure. This analysis used the eligible cohort before the application of the 30-day landmark exclusions and therefore retained patients who initiated outpatient PT after day 30 and those who died or experienced CV-related rehospitalization within 30 days of discharge. Unlike the primary matched-cohort analysis, propensity-score matching was not applied in this sensitivity analysis. Time zero was defined as the date of discharge from the hospital. Patients contributed unexposed person-time from discharge until the date of their first documented outpatient PT session and exposed person-time thereafter. Patients who never initiated outpatient PT remained unexposed throughout the follow-up period. Thus, patients who died before PT initiation contributed their entire observed person-time and death events during the unexposed risk periods. As the registry did not contain session-level dates, daily treatment status, or reliable dates of treatment discontinuation, treatment duration and time-varying treatment intensity could not be incorporated. Accordingly, a once-initiated, always-exposed approach was adopted. The time-dependent Cox model was adjusted for the same prespecified baseline covariates included in the propensity-score model. The model was fitted separately in each of the 50 imputed datasets, and the model coefficients and standard errors were pooled using Rubin’s rule [23]. Follow-up continued until death, censoring, or 1 year after discharge, whichever occurred first.

Kaplan–Meier survival curves were constructed for all outcomes, and group differences were assessed using the log-rank test. Prespecified subgroup analyses were conducted across key clinical characteristics, including CKD stage, to explore whether the association between outpatient PT and outcomes varied across subgroups; effect modification was evaluated using interaction terms. In the overall cohort prior to the eGFR-based exclusion (including CKD stages 1–2), we additionally evaluated the association between CKD stage and 1-year outcomes. The displayed baseline descriptive statistics (Table 1), standardized mean differences, Love plots, Kaplan–Meier curves, and crude event rates were derived from the first representative imputed and matched dataset. All analyses were performed using R software version 4.4.1. A two-sided *p*-value < 0.05 was considered statistically significant.

## 3. Results

### 3.1. Patient Selection and Baseline Characteristics

**Figure 1:**
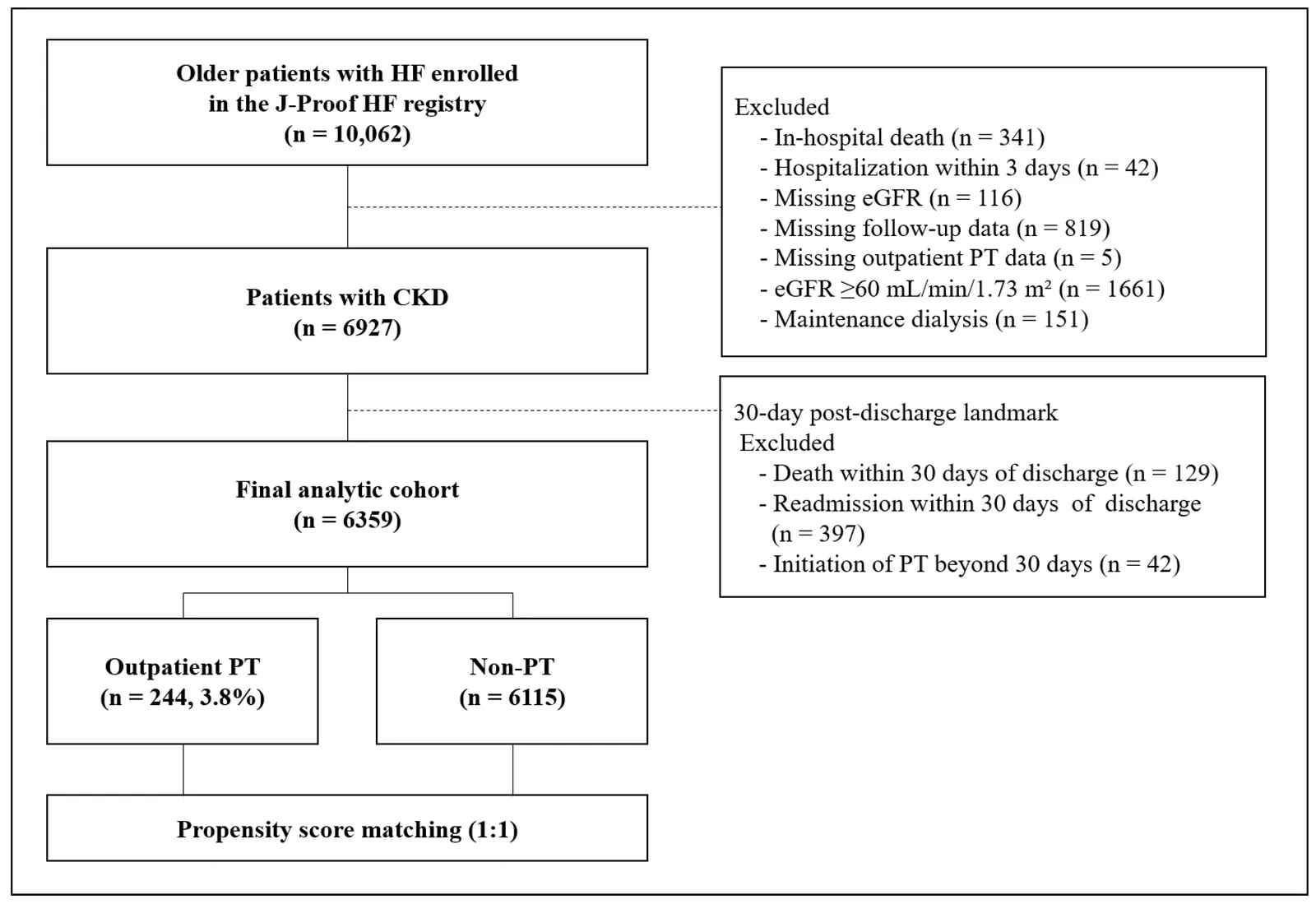
Flow diagram of patient selection. CKD, chronic kidney disease; eGFR, estimated glomerular filtration rate; HF, heart failure; Non-PT, no physical therapy; PT, physical therapy.

In total, 10,062 patients were screened, of whom 6927 had CKD (Figure 1). For the 30-day post-discharge landmark study, the final analytic cohort comprised 6359 patients. Among them, 244 (3.8%) underwent outpatient PT within 30 days of discharge, whereas 6115 had no documented outpatient PT within that period. The highest proportions of missing data were observed for comfortable walking speed (18.0%), grip strength (14.0%), KCL score (11.7%), left atrial diameter (5.5%), serum albumin (2.6%), Barthel Index at discharge (2.4%), BNP (2.0%), and body mass index (1.5%), whereas missingness was below 0.5% for all remaining variables (Online Table S1).

Table 1 shows the baseline characteristics of the groups before and after PSM. Before matching, patients in the PT group were younger (mean age, 77.1 vs. 83.0 years; *p* < 0.001) and had a higher prevalence of HFrEF (59.4% vs. 50.5%, *p* = 0.008), diabetes mellitus (45.1% vs. 35.1%, *p* = 0.002), and dyslipidemia (41.4% vs. 32.5%, *p* = 0.004) than those in the non-PT group. In contrast, hypertension (62.7% vs. 70.4%, *p* = 0.012) and frailty (45.9% vs. 65.6%, *p* < 0.001) were less prevalent in the PT group. The use of ACE inhibitors, angiotensin II receptor blockers, or angiotensin receptor–neprilysin inhibitors (66.0% vs. 54.8%, *p* < 0.001), β-blockers (77.9% vs. 66.2%, *p* < 0.001), and SGLT2 inhibitors (29.9% vs. 15.9%, *p* < 0.001) was more prevalent at discharge among patients who underwent PT.

**Figure 2:**
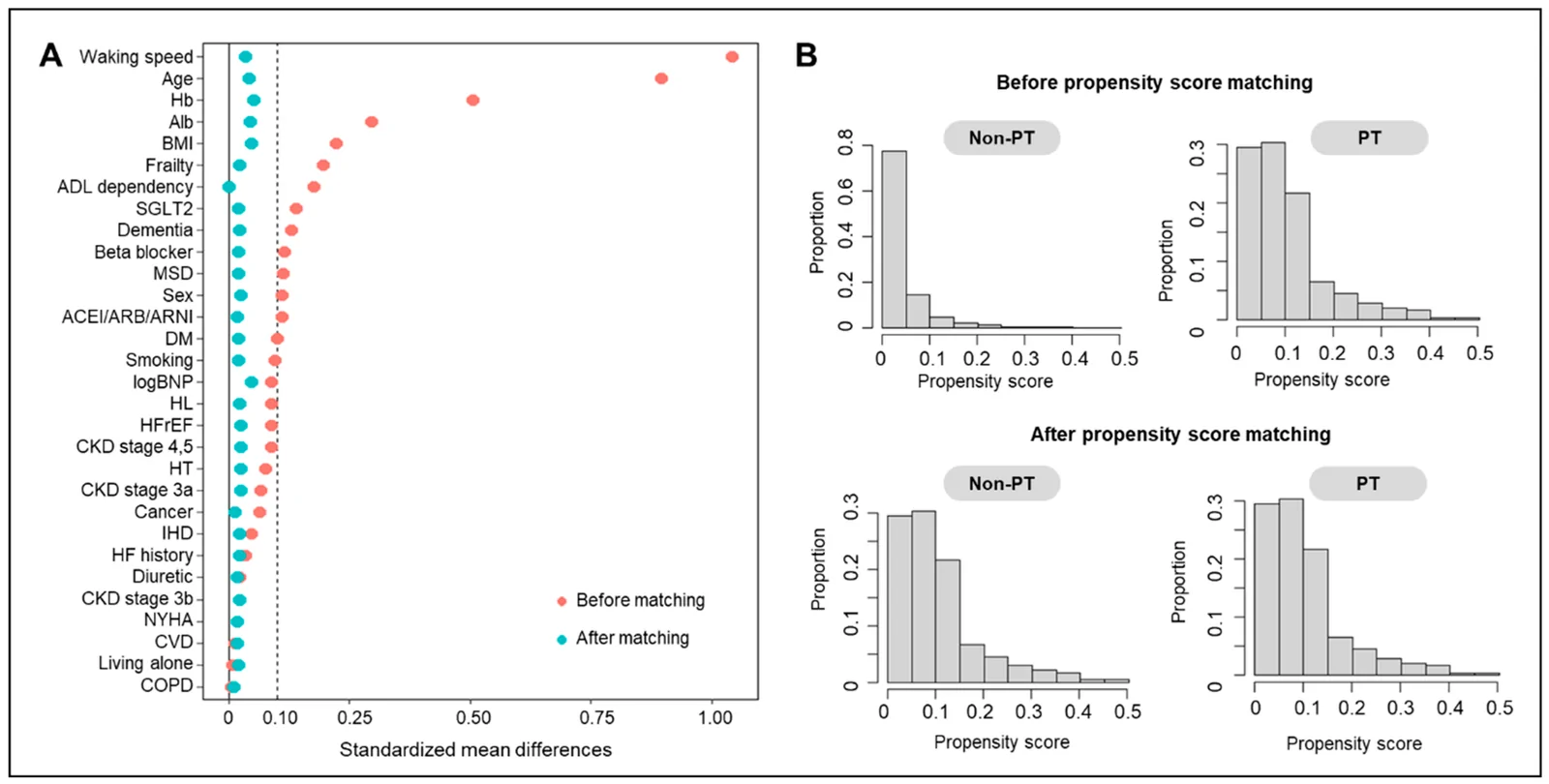
Standardized mean differences and proportions of propensity scores before and after matching. (**A**) Standardized mean differences for baseline demographic, clinical, functional, laboratory, and medication variables before and after 1:1 propensity score matching. (**B**) Proportion of propensity scores before and after matching in the physical therapy and non-physical therapy groups. ACEI, angiotensin-converting enzyme inhibitor; ADL, activities of daily living; Alb, serum albumin; ARB, angiotensin II receptor blocker; ARNI, angiotensin receptor–neprilysin inhibitor; BMI, body mass index; CKD, chronic kidney disease; COPD, chronic obstructive pulmonary disease; CVD, cerebrovascular disease; DM, diabetes mellitus; Hb, hemoglobin; HF, heart failure; HFrEF, heart failure with reduced ejection fraction; HL, dyslipidemia; HT, hypertension; IHD, ischemic heart disease; logBNP, logarithmically transformed B-type natriuretic peptide; MSD, musculoskeletal disease; Non-PT, no physical therapy; NYHA, New York Heart Association class; PT, physical therapy; SGLT2, sodium–glucose cotransporter 2 inhibitor.

PSM yielded 244 pairs of patients. Baseline characteristics were well balanced between groups, with standardized mean differences <0.1 for all variables (Figure 2). No significant differences were observed between the groups after matching.

### 3.2. Follow-Up and Event Rates

During the 1-year post-discharge analysis window, 1099 participants died, 1586 were rehospitalized for CV-related reasons, and 2325 experienced either outcome. In the representative propensity score-matched dataset, 10 patients in the PT group and 21 in the non-PT group died during follow-up; the corresponding crude event proportions were 4.1% and 8.6%, respectively. CV rehospitalization occurred in 55 patients (22.5%) in the PT group and 67 (27.5%) in the non-PT group; the composite outcome occurred in 58 (23.8%) and 82 patients (33.6%), respectively.

### 3.3. Association of PT with Clinical Outcomes

**Figure 3:**
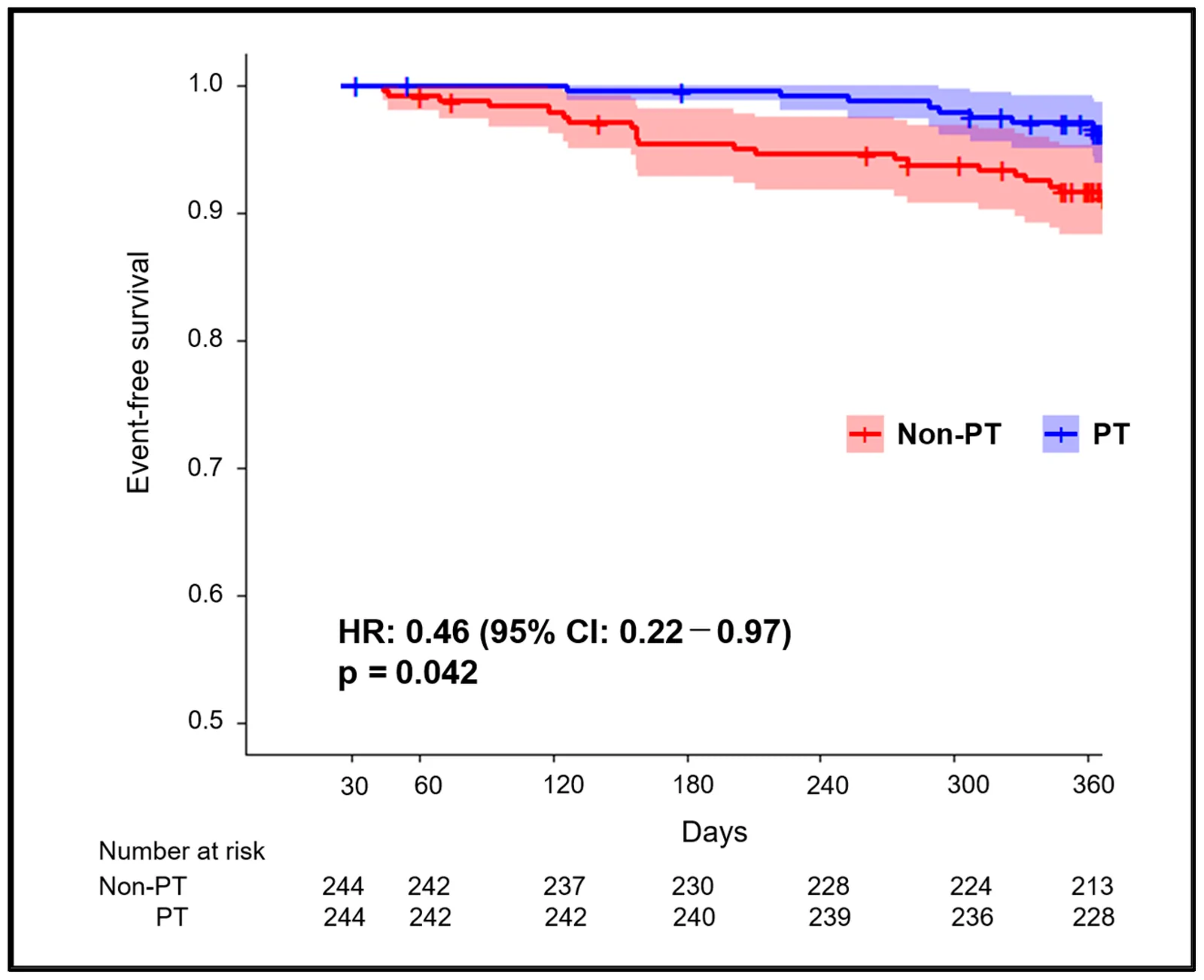
Kaplan–Meier curves for all-cause mortality in the propensity score-matched cohort. In the prespecified landmark design, patients entered the risk set at day 30 after discharge; therefore, the numbers at risk are shown from day 30 onward. The shaded areas represent the 95% CIs. HRs, 95% CIs, and *p*-values were derived from Cox proportional hazards models fitted within the propensity score-matched cohorts, with estimates pooled across the imputed and matched datasets using Rubin’s rules. CI, confidence interval; HR, hazard ratio; non-PT, no physical therapy; PT, physical therapy.

**Figure 4:**
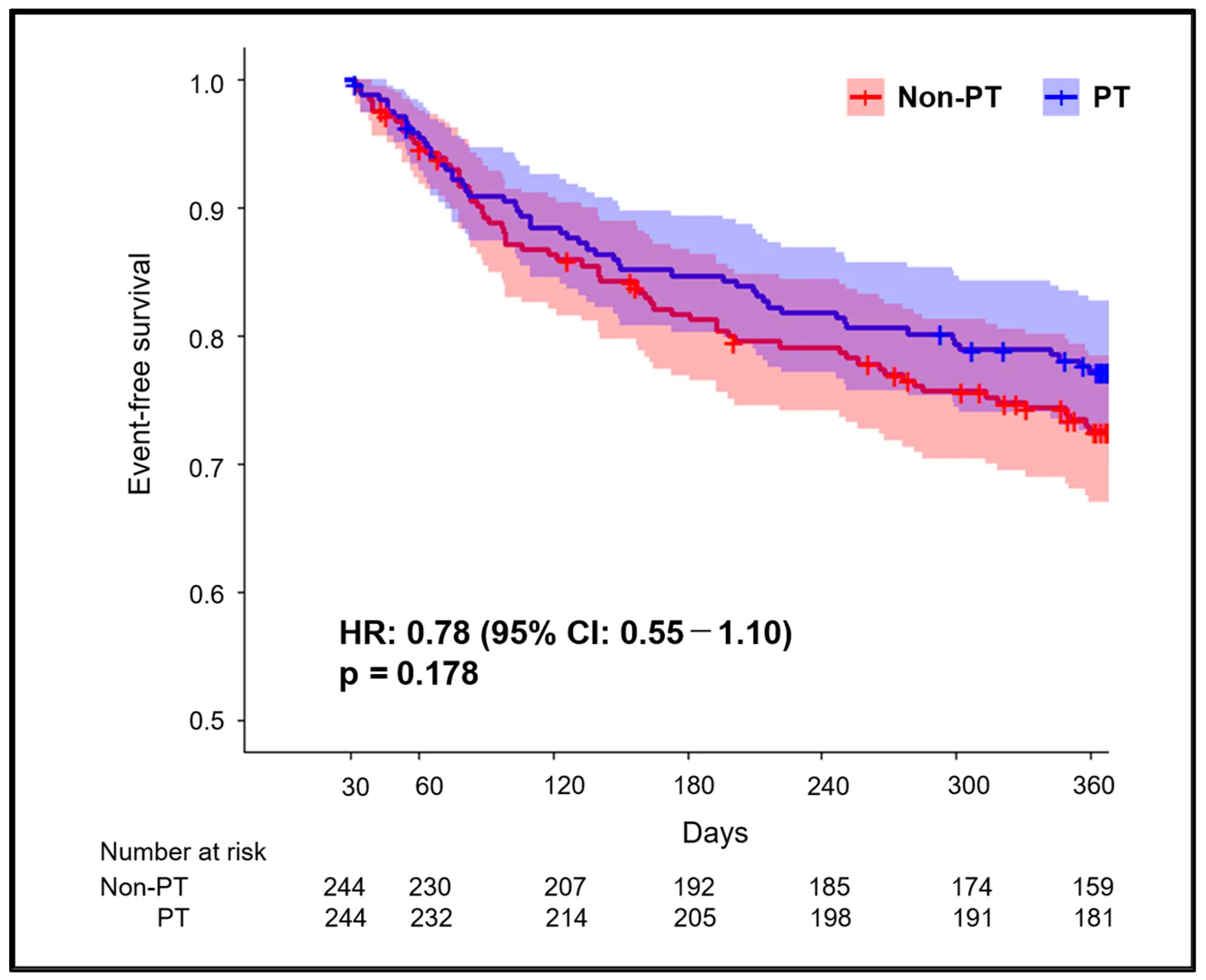
Kaplan–Meier curves for cardiovascular-related rehospitalization in the propensity score-matched cohort. In the prespecified landmark design, patients entered the risk set at day 30 after discharge; therefore, the numbers at risk are shown from day 30 onward. The shaded areas represent the 95% CIs. HRs, 95% CIs, and *p*-values were derived from Cox proportional hazards models fitted within the propensity score-matched cohorts, with estimates pooled across the imputed and matched datasets using Rubin’s rules. CI, confidence interval; HR, hazard ratio; non-PT, no physical therapy; PT, physical therapy.

**Figure 5:**
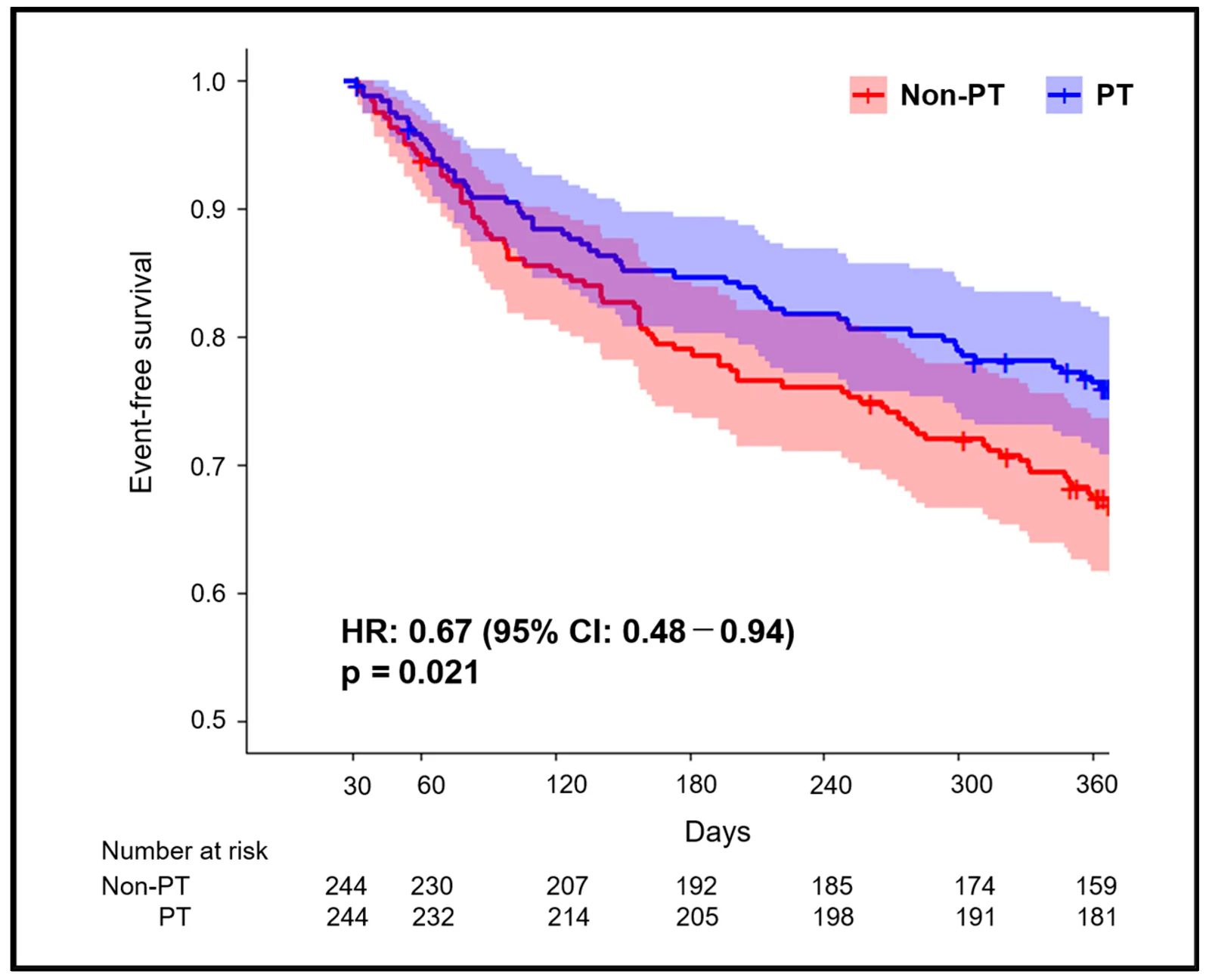
Kaplan–Meier curves for the composite outcome of all-cause mortality or cardiovascular-related rehospitalization in the propensity score-matched cohort. In the prespecified landmark design, patients entered the risk set at day 30 after discharge; therefore, the numbers at risk are shown from day 30 onward. The shaded areas represent the 95% CIs. HRs, 95% CIs, and *p*-values were derived from Cox proportional hazards models fitted within the propensity score-matched cohorts, with estimates pooled across the imputed and matched datasets using Rubin’s rules. CI, confidence interval; HR, hazard ratio; non-PT, no physical therapy; PT, physical therapy.

In Cox regression analyses of the matched cohorts, early outpatient PT participation was associated with a lower hazard of 1-year all-cause mortality compared with no documented outpatient PT participation (hazard ratio [HR], 0.46; 95% confidence interval [CI], 0.22–0.97; *p* = 0.042) (Figure 3). Early outpatient PT participation was not significantly associated with the hazard of CV rehospitalization (HR, 0.78; 95% CI, 0.55–1.10; *p* = 0.178) (Figure 4). Consistent with this finding, the Fine–Gray competing-risk analysis, in which death was treated as a competing event, showed no statistically significant association between outpatient PT participation and CV rehospitalization (subdistribution HR, 0.93; 95% CI, 0.59–1.46; *p* = 0.739). Early outpatient PT participation was associated with a lower hazard of the composite outcome of all-cause mortality or CV rehospitalization (HR, 0.67; 95% CI, 0.48–0.94; *p* = 0.021) (Figure 5).

In the time-dependent Cox sensitivity analysis using the eligible cohort before the application of the 30-day landmark exclusions, patients who initiated outpatient PT after day 30 were retained and contributed unexposed person-time before PT initiation and exposed person-time thereafter. Outpatient PT initiation was associated with a lower hazard of all-cause mortality in the adjusted time-dependent Cox model (adjusted HR, 0.35; 95% CI, 0.18–0.67; *p* = 0.001).

### 3.4. Subgroup and Supplementary Analyses

The results of subgroup analyses are summarized in Online Tables S2–S4. Subgroup estimates were imprecise, with wide CIs and sparse events in several strata. These exploratory analyses were underpowered to establish effect homogeneity or heterogeneity. For the overall cohort prior to eGFR-based exclusion (thus including patients with CKD stages 1–2), CKD stage-stratified event curves were constructed for 1-year outcomes. These are presented in Online Figure 1, Figure 2 and Figure 3.

## 4. Discussion

### 4.1. Principal Findings and Relevance to Older Patients with HF

In this retrospective nationwide multicenter registry study, a 30-day post-discharge landmark cohort of 6359 older patients hospitalized for worsening HF who met the study-defined CKD criterion was analyzed. Among these 30-day event-free survivors, 3.8% underwent outpatient PT within 30 days of discharge. Despite this low uptake, participation in PT was associated with a significantly lower 1-year risk of all-cause mortality, whereas CV-related rehospitalization alone was not statistically significant. However, the composite endpoint of mortality or CV-related rehospitalization was significantly lower among patients undergoing PT within 30 days of discharge than among those who did not undergo PT. These findings support evidence that exercise-based rehabilitation improves functional capacity and health-related quality of life in patients with HF, while effects on hard outcomes vary according to population, intervention content, and adherence [24,25,26,27]. This study provides real-world data in an older HF–CKD population, a group with limited trial evidence and substantial implementation barriers.

Importantly, the PSM cohort had very high ADL function, suggesting that the matched sample reflected characteristics of patients who underwent outpatient PT within 30 days of discharge. Accordingly, these findings should be interpreted as most applicable to older patients with HF and CKD who have a preserved functional status, and caution is warranted when generalizing to patients with substantial ADL dependence.

### 4.2. Biological Plausibility Across Aging, HF, and CKD

The association between PT participation and lower mortality is biologically plausible in patients with HF complicated by CKD, in which chronic inflammation, oxidative stress, endothelial dysfunction, autonomic imbalance, anemia and iron dysregulation, mitochondrial dysfunction, and impaired skeletal muscle energetics converge to reduce physiological reserve. Exercise in CKD benefits vascular, metabolic, autonomic, and skeletal muscle pathways beyond cardiorespiratory fitness [28], and aerobic exercise has been shown to reduce oxidative stress biomarkers [29]. The heart–kidney interaction framework suggests that interventions enhancing peripheral conditioning and self-management may be associated with improved survival, even without clearly reducing CV-related rehospitalizations [8,30,31], particularly in older adults with a limited homeostatic reserve.

From a geriatric perspective, even modest improvements in mobility, strength, balance, and endurance can markedly enhance resilience and independence. Frailty and disability trajectories strongly predict outcomes in later life [32,33,34,35,36], and within an ADL-independent population, preserving functional reserve during the vulnerable post-discharge phase may represent an important prognostic factor and potential target for future interventions.

### 4.3. Associations with Mortality vs. CV-Related Rehospitalization

A notable pattern in our results was that PT initiated within 30 days after discharge was significantly associated with all-cause mortality and the composite endpoint but not with CV-related rehospitalization alone. Several mechanisms may explain this dissociation, particularly in older patients with HF.

First, the observed association between PT participation and lower mortality may reflect pathways related to systemic vulnerability that contribute to mortality—such as frailty progression, falls, infections, malnutrition, and catabolic decline—rather than directly preventing CV-related rehospitalizations [32,33,34,35,36]. In older adults, survival is often determined by resilience to diverse stressors rather than by the targeted prevention of single-organ events.

Second, early readmissions and adverse outcomes after HF hospitalization are frequently driven by systemic vulnerability rather than by recurrence of the index cardiac pathology alone [37,38], consistent with the concept of post-hospital syndrome, a transient state of generalized risk characterized by physiological stress, deconditioning, and impaired homeostasis [39,40]. The association between PT participation and lower mortality may involve pathways related to reconditioning, functional stability, and self-management behaviors without necessarily eliminating CV-related rehospitalization.

Third, endpoint-specific considerations are relevant. The effect estimate for CV-related rehospitalization was smaller than that for mortality, limiting the study’s power to detect smaller or heterogeneous effects across centers and event subtypes. In addition, the ascertainment of nonfatal CV-related rehospitalization likely varied among the 96 centers included in this study, in contrast with that of all-cause mortality, potentially attenuating observable associations.

### 4.4. CKD Severity and Consistency with Prior CR Evidence

In the subgroup analyses, point estimates varied across kidney-function strata, and the analyses were not adequately powered to establish homogeneity or heterogeneity of the association. For CV-related rehospitalization and the composite outcome, point estimates were <1.0 across CKD stages 3a, 3b, and 4–5. For all-cause mortality, no estimate could be determined for patients with CKD stage 3a because no deaths occurred in the PT group, whereas the point estimates for CKD stages 3b and 4 to 5 were below 1.0. Formal tests for PT-by-CKD interaction were not statistically significant for any outcome; however, because the subgroup estimates were imprecise and several strata contained sparse events, these underpowered analyses do not establish either homogeneity or heterogeneity across CKD stages.

Conversely, Hamazaki et al. [41] reported CKD-stratified differences in outpatient CR outcomes, suggesting that benefits may decrease with worsening kidney function. Given that renal dysfunction strongly predicts prognosis in HF, this result is plausible. In this study, however, CKD-stratified estimates were imprecise, with wide CIs and sparse events in some strata, limiting our ability to definitively assess stage-dependent differences in the effect size. Larger studies with greater event counts are needed to determine whether CKD severity modifies the magnitude of the observed PT/CR–outcome associations. Meanwhile, CKD-aware program adaptation and careful risk management remain essential for the safe and feasible implementation of PT in clinical practice [28,42].

### 4.5. Clinical and Health-System Implications in a Rapidly Aging Society

Despite guideline endorsement of exercise-based rehabilitation in HF [24,25,26,27], outpatient PT uptake was strikingly low in this cohort. This likely reflects real-world barriers in older patients with CKD, including frailty, transportation difficulties, and competing medical demands. One kidney-care framework emphasized multidisciplinary risk management and identified physical inactivity as a modifiable target, underscoring the need for rehabilitation strategies that can be adapted according to a patient’s kidney function and age [42].

In this context, scalable delivery models that lower access barriers—such as home-based, hybrid, and tele-rehabilitation approaches—represent pragmatic alternatives to conventional outpatient programs [43,44,45]. The evaluation of such models warrants prioritization in aging societies that face a growing HF burden [2], particularly in policy-relevant settings such as Japan’s long-term care system [46].

Notably, participation in outpatient PT—reflecting early engagement rather than treatment intensity—was associated with favorable outcomes in this study. This observation should be interpreted cautiously, as it may reflect early engagement with post-discharge care, patient motivation, or residual confounding rather than a direct dose–response effect of PT itself. Nevertheless, it raises the possibility that early post-discharge contact with rehabilitation services, irrespective of delivery format, may function as an important marker of favorable care trajectories.

### 4.6. Limitations and Methodological Considerations

Some limitations warrant consideration. First, because this was an observational study, residual confounding could not be fully excluded. A key strength was the use of PSM to construct a comparison cohort with closely balanced baseline characteristics, reducing confounding by indication. To further address the potential immortal time bias, we performed a time-dependent Cox regression analysis in which outpatient PT status was modeled as a time-varying exposure according to the timing of PT initiation after discharge. In this sensitivity analysis, using the eligible cohort before the application of the 30-day landmark exclusions, patients who initiated outpatient PT after day 30 were retained and contributed unexposed person-time before PT initiation and exposed person-time thereafter. Outpatient PT initiation was associated with a lower hazard of all-cause mortality in the adjusted time-dependent Cox model. Although this analysis reduces concerns regarding the misclassification of pre-initiation person-time, it does not exclude time-dependent confounding, treatment-selection bias, or other residual sources of bias; therefore, it does not establish causality. We also mitigated time-related bias using a 30-day landmark design, excluding early deaths and restricting PT initiation, in line with guidance on immortal time bias and target trial emulation [47,48]. We used multiple imputation for missing data and propensity score matching for measured baseline confounding [23,49,50]. Because matching achieved acceptable covariate balance and the number of mortality events was limited, no additional covariate adjustment was performed in the matched-cohort outcome models. Although PSM improved balance in measured baseline covariates, substantial treatment-selection mechanisms may remain. Patients who participated in outpatient PT were likely to differ from nonparticipants in unmeasured characteristics such as motivation, transportation, healthcare access, social support, cognition, post-discharge recovery, and clinician referral. Therefore, healthy-user bias, confounding by indication, and residual confounding cannot be excluded, and the observed association should not be interpreted as a causal effect of PT.

Second, PT exposure was defined pragmatically as participation in at least one PT session. Although cumulative session counts were available descriptively, session-level timing, frequency, intensity, adherence, and reasons for discontinuation were unavailable; therefore, the session counts could not support a reliable causal dose–response analysis. Third, although we accounted for ADL dependency in adjusting for potential confounders, the PSM cohort consisted predominantly of patients with a preserved ADL function at discharge, which may limit the generalizability of our results to more disabled populations. Fourth, heterogeneity in the ascertainment of CV-related rehospitalization across centers might have attenuated the association between outpatient PT and this non-fatal endpoint.

Some patients classified as having CKD may instead have had acute or transient kidney dysfunction related to congestion, impaired renal perfusion, diuretic therapy, acute cardiorenal deterioration, or systemic comorbidities, including hepatic dysfunction and liver cirrhosis [51]. Accordingly, the findings should be interpreted as applying to patients who met the study-defined eGFR criterion for CKD during hospitalization. Additionally, all participants included in this registry analysis had received PT during the index hospitalization; therefore, the findings may not be generalizable to the overall population of patients with HF and CKD, particularly those not referred for inpatient PT, those with severe disability, or those treated in settings with different PT pathways. Finally, our subgroup analyses according to CKD stage were underpowered and must be considered exploratory.

## 5. Conclusions

In this secondary analysis of a nationwide registry of older patients with HF and study-defined CKD who were alive and free from CV rehospitalization at the 30-day landmark, early outpatient PT participation was associated with a lower risk of subsequent all-cause mortality, whereas no statistically significant association with CV rehospitalization was demonstrated. These findings apply to selected 30-day event-free survivors and warrant cautious interpretation given the observational study design. Further prospective controlled studies are needed to determine whether the observed association reflects a causal effect of outpatient PT on survival.

## Figures and Tables

**Table 1:** Baseline characteristics before and after propensity score matching.

	Before Propensity Score Matching			After Propensity Score Matching		
	Non-PT	PT	*p* Value	Non-PT	PT	*p* Value
	*n* = 6115	*n* = 244		*n* = 244	*n* = 244	
Age, years, mean (SD)	83.0 (7.5)	77.1 (6.6)	<0.001	76.9 (7.7)	77.1 (6.6)	0.767
Male sex, *n* (%)	3078 (50.3)	150 (61.5)	0.001	158 (64.8)	150 (61.5)	0.511
Body mass index, kg/m^2^, mean (SD)	22.7 (4.9)	23.6 (4.4)	0.003	23.9 (5.1)	23.6 (4.4)	0.462
Current smoker, *n* (%)	783 (12.8)	55 (22.5)	<0.001	64 (26.2)	55 (22.5)	0.399
NYHA functional class, *n* (%)						
I	218 (3.6)	8 (3.3)	0.831	11 (4.5)	8 (3.3)	0.649
II	1156 (18.9)	42 (17.2)		44 (18.0)	42 (17.2)	
III	2532 (41.4)	99 (40.6)		107 (43.9)	99 (40.6)	
IV	2199 (36.0)	95 (38.9)		82 (33.6)	95 (38.9)	
History of heart failure, *n* (%)						
None	3588 (58.7)	152 (62.3)	0.016	146 (59.8)	152 (62.3)	0.159
>1 year before	1769 (28.9)	52 (21.3)		68 (27.9)	52 (21.3)	
≤1 year before	758 (12.4)	40 (16.4)		30 (12.3)	40 (16.4)	
Chronic kidney disease stage, *n* (%)						
3a	1901 (31.1)	92 (37.7)	0.010	91 (37.3)	92 (37.7)	0.951
3b	2170 (35.5)	92 (37.7)		90 (36.9)	92 (37.7)	
4–5	2044 (33.4)	60 (24.6)		63 (25.8)	60 (24.6)	
Comorbidities, *n* (%)						
Hypertension	4307 (70.4)	153 (62.7)	0.012	167 (68.4)	153 (62.7)	0.216
Diabetes mellitus	2145 (35.1)	110 (45.1)	0.002	122 (50.0)	110 (45.1)	0.319
Dyslipidemia	1985 (32.5)	101 (41.4)	0.004	114 (46.7)	101 (41.4)	0.274
Chronic obstructive pulmonary disease	431 (7.0)	16 (6.6)	0.868	15 (6.1)	16 (6.6)	>0.99
Cerebrovascular disease	964 (15.8)	35 (14.3)	0.611	41 (16.8)	35 (14.3)	0.532
Cancer	1019 (16.7)	25 (10.2)	0.010	21 (8.6)	25 (10.2)	0.642
Musculoskeletal disorders	1643 (26.9)	38 (15.6)	<0.001	35 (14.3)	38 (15.6)	0.800
Dementia	2395 (39.2)	61 (25.0)	<0.001	53 (21.7)	61 (25.0)	0.456
Peripheral arterial disease	374 (6.1)	13 (5.3)	0.784	14 (5.7)	13 (5.3)	>0.99
Etiology of heart failure, *n* (%)						
Ischemic heart disease	1840 (30.1)	62 (25.4)	0.135	66 (27.0)	62 (25.4)	0.758
Valvular heart disease	2368 (38.7)	75 (30.7)	0.014	76 (31.1)	75 (30.7)	>0.99
Cardiomyopathy	634 (10.4)	55 (22.5)	<0.001	44 (18.0)	55 (22.5)	0.260
Arrhythmia	3057 (50.0)	137 (56.1)	0.069	127 (52.0)	137 (56.1)	0.414
Echocardiography, mean (SD)						
LVEF, %	47.8 (16.4)	44.4 (17.2)	0.001	42.8 (17.5)	44.4 (17.2)	0.317
Left atrial diameter, mm	44.7 (9.0)	45.0 (10.0)	0.659	46.1 (9.6)	45.0 (10.0)	0.379
HFrEF, *n* (%)	3088 (50.5)	145 (59.4)	0.008	154 (63.1)	145 (59.4)	0.457
Blood biochemistry tests						
BNP, pg/mL, median [Q1, Q3]	576.1 [326.0, 1025.2]	525.4 [267.2, 884.2]	0.123	542.9 [342.4, 1038.2]	525.4 [267.2, 884.2]	0.208
Serum creatinine, mg/dL, mean (SD)	1.53 (0.90)	1.48 (0.82)	0.336	1.44 (0.63)	1.48 (0.82)	0.618
eGFR, mL/min/1.73 m^2^, mean (SD)	36.3 (13.5)	38.8 (12.7)	0.004	38.9 (12.8)	38.8 (12.7)	0.942
BUN, mg/dL, mean (SD)	31.2 (16.2)	28.2 (14.0)	0.005	27.9 (14.2)	28.2 (14.0)	0.824
Serum albumin, g/dL, mean (SD)	3.5 (0.5)	3.6 (0.5)	<0.001	3.6 (0.5)	3.7 (0.5)	0.672
Hemoglobin, g/dL, mean (SD)	11.3 (2.2)	12.5 (2.2)	<0.001	12.5 (2.5)	12.5 (2.2)	0.774
Sodium, mmol/L, mean (SD)	139.7 (4.3)	139.2 (4.2)	0.104	139.6 (4.2)	139.2 (4.2)	0.335
Medication use, *n* (%)						
ACE inhibitor/ARB/ARNI	3354 (54.8)	161 (66.0)	0.001	174 (71.3)	161 (66.0)	0.242
Mineralocorticoid receptor antagonist	3327 (54.4)	171 (70.1)	<0.001	164 (67.2)	171 (70.1)	0.558
β-blocker	4048 (66.2)	190 (77.9)	<0.001	192 (78.7)	190 (77.9)	0.913
SGLT2 inhibitor	970 (15.9)	73 (29.9)	<0.001	81 (33.2)	73 (29.9)	0.495
Diuretic	5255 (85.9)	204 (83.6)	0.336	207 (84.8)	204 (83.6)	0.804
Kihon Checklist score, median [Q1, Q3]	10.00 [4.00, 14.00]	7.50 [4.00, 11.00]	<0.001	7.00 [2.00, 11.00]	7.50 [4.00, 11.00]	0.096
Frailty, *n* (%)	4010 (65.6)	112 (45.9)	<0.001	108 (44.3)	112 (45.9)	0.785
Living alone, *n* (%)	1626 (26.6)	63 (25.8)	0.847	67 (27.5)	63 (25.8)	0.759
Barthel Index at discharge, mean (SD)	82 (23)	98 (5)	<0.001	96 (7)	98 (5)	0.121
Grip strength, kg, mean (SD)	16.1 (9.7)	22.3 (9.3)	<0.001	21.6 (10.8)	22.3 (9.3)	0.449
Comfortable walking speed, m/s, mean (SD)	0.74 (0.27)	0.98 (0.25)	<0.001	0.94 (0.24)	0.98 (0.25)	0.347
Number of outpatient physical therapy sessions, *n* (%)						
0 sessions	6115 (100)	0 (0)		244 (100)	0 (0)	
1–4 sessions	0 (0)	60 (24.6)		0 (0)	60 (24.6)	
5–9 sessions	0 (0)	48 (19.7)		0 (0)	48 (19.7)	
≥10 sessions	0 (0)	136 (55.7)		0 (0)	136 (55.7)	
Outcome						
All-cause mortality	1089 (17.8)	10 (4.1)		21 (8.6)	10 (4.1)	
CV rehospitalization	1531 (25.0)	55 (22.5)		67 (27.5)	55 (22.5)	
Composite outcome	2267 (37.1)	58 (23.8)		82 (33.6)	58 (23.8)	

ACE, angiotensin-converting enzyme; ARB, angiotensin II receptor blocker; ARNI, angiotensin receptor–neprilysin inhibitor; BNP, B-type natriuretic peptide; BUN, blood urea nitrogen; CV, cardiovascular; eGFR, estimated glomerular filtration rate; HFrEF, heart failure with reduced ejection fraction; LVEF, left ventricular ejection fraction; Non-PT, no physical therapy; NYHA, New York Heart Association; PT, physical therapy; Q, quartile; SD, standard deviation; SGLT2, sodium–glucose cotransporter 2.

## Data Availability

Deidentified participant data will not be shared because of restrictions related to patient privacy and informed consent.

## Supplementary Materials

The following supporting information can be downloaded at https://www.mdpi.com/article/10.3390/jcm15187043/s1, Figure S1: Association between CKD Stage and All-cause Mortality in Older Patients with Heart Failure, Figure S2: Association between CKD Stage and Cardiovascular Rehospitalization in Older Patients with Heart Failure, Figure S3: Association between CKD Stage and Composite Outcome of All-Cause Mortality and Cardiovascular Rehospitalization in Older Patients with Heart Failure, Table S1: Missing data summary for baseline variables in the full study cohort; Table S2: Subgroup analyses of the association between post-discharge physical therapy and all-cause mortality; Table S3: Subgroup analyses of the association between post-discharge physical therapy and cardiovascular-related rehospitalization; Table S4: Subgroup analyses of the association between post-discharge physical therapy and the composite outcome of all-cause mortality or cardiovascular-related rehospitalization.

## Abbreviations

The following abbreviations are used in this manuscript:

ACE	angiotensin-converting enzyme
ADL	activities of daily living
BNP	B-type natriuretic peptide
CI	confidence interval
CKD	chronic kidney disease
CR	cardiac rehabilitation
CV	cardiovascular
eGFR	estimated glomerular filtration rate
HF	heart failure
HFrEF	heart failure with reduced ejection fraction
HR	hazard ratio
KCL	Kihon Checklist
LVEF	left ventricular ejection fraction
NT-proBNP	N-terminal pro-B-type natriuretic peptide
NYHA	New York Heart Association
PSM	propensity score matching
PT	physical therapy
SGLT2	sodium–glucose cotransporter 2
t0	time zero
UMIN	University Hospital Medical Information Network

## References

[1] Statistics Bureau of Japan Population Statistics 2024. https://www.stat.go.jp/english/data/jinsui/2024np/index.html.

[2] Shimokawa H., Miura M., Nochioka K., Sakata Y. (2015). Heart failure as a general pandemic in Asia. Eur. J. Heart Fail..

[3] Conrad N., Judge A., Tran J., Mohseni H., Hedgecott D., Crespillo A.P., Allison M., Hemingway H., Cleland J.G., McMurray J.J. (2018). Temporal trends and patterns in heart failure incidence: A population-based study of 4 million individuals. Lancet.

[4] Vigen R., Maddox T.M., Allen L.A. (2012). Aging of the United States population: Impact on heart failure. Curr. Heart Fail. Rep..

[5] Damman K., Valente M.A.E., Voors A.A., O’Connor C.M., van Veldhuisen D.J., Hillege H.L. (2014). Renal impairment, worsening renal function, and outcome in patients with heart failure: An updated meta-analysis. Eur. Heart J..

[6] Gerhardt L.M.S., Kordsmeyer M., Sehner S., Güder G., Störk S., Edelmann F., Wachter R., Pankuweit S., Prettin C., Ertl G. (2023). Prevalence and prognostic impact of chronic kidney disease and anaemia across ACC/AHA precursor and symptomatic heart failure stages. Clin. Res. Cardiol..

[7] Denic A., Glassock R.J., Rule A.D. (2016). Structural and functional changes with the aging kidney. Adv. Chronic Kidney Dis..

[8] Rangaswami J., Bhalla V., Blair J.E.A., Chang T.I., Costa S., Lentine K.L., Lerma E.V., Mezue K., Molitch M., Mullens W. (2019). Cardiorenal syndrome: Classification, pathophysiology, diagnosis, and treatment strategies: A scientific statement from the American Heart Association. Circulation.

[9] Secora A., Alexander G.C., Ballew S.H., Coresh J., Grams M.E. (2018). Kidney function, polypharmacy, and potentially inappropriate medication use in a community-based cohort of older adults. Drugs Aging.

[10] Österman J., Al-Sodany E., Haugen Löfman I., Barany P., Evans M. (2024). Heart failure: The grim reaper of the cardio-renal-metabolic triad. ESC Heart Fail..

[11] Tang W.H.W., Bakitas M.A., Cheng X.S., Fang J.C., Fedson S.E., Fiedler A.G., Martens P., Mccallum W.I., Ogunniyi M.O., Rangaswami J. (2024). Evaluation and management of kidney dysfunction in advanced heart failure: A scientific statement from the American Heart Association. Circulation.

[12] Makita S., Yasu T., Akashi Y.J., Adachi H., Izawa H., Ishihara S., Iso Y., Ohuchi H., Omiya K., Ohya Y. (2023). JCS/JACR 2021 guideline on rehabilitation in patients with cardiovascular disease. Circ. J..

[13] McDonagh T.A., Metra M., Adamo M., Gardner R.S., Baumbach A., Böhm M., Burri H., Butler J., Čelutkienė J., Chioncel O. (2021). 2021 ESC Guidelines for the diagnosis and treatment of acute and chronic heart failure. Eur. Heart J..

[14] Brown T.M., Pack Q.R., Aberegg E., Brewer L.C., Ford Y.R., Forman D.E., Gathright E.C., Khadanga S., Ozemek C., Thomas R.J. (2024). Core components of cardiac rehabilitation programs: 2024 update: A scientific statement from the American Heart Association and the American Association of Cardiovascular and Pulmonary Rehabilitation. Circulation.

[15] Molloy C., Long L., Mordi I.R., Bridges C., Sagar V.A., Davies E.J., Coats A.J., Dalal H., Rees K., Singh S.J. (2024). Exercise-based cardiac rehabilitation for adults with heart failure. Cochrane Database Syst. Rev..

[16] Kitzman D.W., Whellan D.J., Duncan P., Pastva A.M., Mentz R.J., Reeves G.R., Nelson M.B., Chen H., Upadhya B., Reed S.D. (2021). Physical rehabilitation for older patients hospitalized for heart failure. N. Engl. J. Med..

[17] Kamiya K., Sato Y., Takahashi T., Tsuchihashi-Makaya M., Kotooka N., Ikegame T., Takura T., Yamamoto T., Nagayama M., Goto Y. (2020). Multidisciplinary cardiac rehabilitation and long-term prognosis in patients with heart failure. Circ. Heart Fail..

[18] Takahashi T., Iwata K., Morisawa T., Kato M., Kono Y., Taya M., Iida Y., Funami Y., Kamiya K., Sakurada K. (2024). Incidence of hospitalization-associated disability in older patients with heart failure. Circ. J..

[19] Ishihara S., Hiramitsu S., Kanaoka K., Taki M., Nakagawa H., Ueda T., Seno A., Nishida T., Onoue K., Soeda T. (2022). New conversion formula between B-type natriuretic peptide and N-terminal-pro-B-type natriuretic peptide—Analysis from a multicenter study. Circ. J..

[20] Satake S., Senda K., Hong Y.J., Miura H., Endo H., Sakurai T., Kondo I., Toba K. (2016). Validity of the Kihon Checklist for assessing frailty status. Geriatr. Gerontol. Int..

[21] JCS Joint Working Group (2014). Guidelines for rehabilitation in patients with cardiovascular disease (JCS 2012). Circ. J..

[22] (2024). Kidney Disease: Improving Global Outcomes (KDIGO) CKD Work Group. KDIGO 2024 Clinical Practice Guideline for the Evaluation and Management of Chronic Kidney Disease. Kidney Int..

[23] Rubin D.B. (2004). Multiple Imputation for Nonresponse in Surveys.

[24] Molloy C.D., Long L., Mordi I.R., Bridges C., Sagar V.A., Davies E.J., Coats A.J., Dalal H., Rees K., Singh S.J. (2023). Exercise-based cardiac rehabilitation for adults with heart failure—2023 Cochrane systematic review and meta-analysis. Eur. J. Heart Fail..

[25] O’Connor C.M., Whellan D.J., Lee K.L., Keteyian S.J., Cooper L.S., Ellis S.J., Leifer E.S., Kraus W.E., Kitzman D.W., Blumenthal J.A. (2009). Efficacy and safety of exercise training in patients with chronic heart failure: HF-ACTION randomized controlled trial. JAMA.

[26] Kitai T., Kohsaka S., Kato T., Kato E., Sato K., Teramoto K., Yaku H., Akiyama E., Ando M., Izumi C. (2025). JCS/JHFS 2025 guideline on diagnosis and treatment of heart failure. J. Card. Fail..

[27] Yamagata K., Hoshino J., Sugiyama H., Hanafusa N., Shibagaki Y., Komatsu Y., Konta T., Fujii N., Kanda E., Sofue T. (2019). Clinical practice guideline for renal rehabilitation: Systematic reviews and recommendations of exercise therapies in patients with kidney diseases. Ren. Replace. Ther..

[28] Bishop N.C., Burton J.O., Graham-Brown M.P.M., Stensel D.J., Viana J.L., Watson E.L. (2023). Exercise and chronic kidney disease: Potential mechanisms underlying the physiological benefits. Nat. Rev. Nephrol..

[29] Zhao M., Xiao M., Tan Q., Lyu J., Lu F. (2023). The effect of aerobic exercise on oxidative stress in patients with chronic kidney disease: A systematic review and meta-analysis with trial sequential analysis. Ren. Fail..

[30] Scrutinio D., Guida P., Carbonara R., Passantino A. (2025). Cardiac rehabilitation for old-old patients with heart failure and severe functional impairment. Int. J. Cardiol..

[31] Baudry G., Pereira O., Duarte K., Ferreira J.P., Savarese G., Welter A., Tangre P., Lamiral Z., Agrinier N., Girerd N. (2024). Risk of readmission and death after hospitalization for worsening heart failure: Role of post-discharge follow-up visits in a real-world study from the Grand Est Region of France. Eur. J. Heart Fail..

[32] Fried L.P., Tangen C.M., Walston J., Newman A.B., Hirsch C., Gottdiener J., Seeman T., Tracy R., Kop W.J., Burke G. (2001). Frailty in older adults: Evidence for a phenotype. J. Gerontol. Ser. A Biol. Sci. Med. Sci..

[33] Ohashi K., Matsue Y., Maeda D., Fujimoto Y., Kagiyama N., Sunayama T., Dotare T., Jujo K., Saito K., Kamiya K. (2024). Impact of multidomain frailty on the mode of death in older patients with heart failure: A cohort study. Circ. Cardiovasc. Qual. Outcomes.

[34] Maeda D., Matsue Y., Kagiyama N., Fujimoto Y., Sunayama T., Dotare T., Nakade T., Jujo K., Kamiya K., Saito H. (2025). Clinical and prognostic utility of the Essential Frailty Toolset in older patients with heart failure. J. Am. Geriatr. Soc..

[35] Zheng P.P., Yao S.M., He W., Wan Y.H., Wang H., Yang J.F. (2021). Frailty related all-cause mortality or hospital readmission among adults aged 65 and older with stage-B heart failure inpatients. BMC Geriatr..

[36] van Seben R., Covinsky K.E., Reichardt L.A., Aarden J.J., van der Schaaf M., van der Esch M., Engelbert R.H.H., Twisk J.W.R., Bosch J.A., Buurman B.M. (2020). Insight into the posthospital syndrome: A 3-month longitudinal follow up on geriatric syndromes and their association with functional decline, readmission, and mortality. J. Gerontol. Ser. A Biol. Sci. Med. Sci..

[37] Dharmarajan K., Hsieh A.F., Lin Z., Bueno H., Ross J.S., Horwitz L.I., Barreto-Filho J.A., Kim N., Bernheim S.M., Suter L.G. (2013). Diagnoses and timing of 30-day readmissions after hospitalization for heart failure, acute myocardial infarction, or pneumonia. JAMA.

[38] Chen Y., Almirall-Sánchez A., Mockler D., Adrion E., Domínguez-Vivero C., Romero-Ortuño R. (2022). Hospital-associated deconditioning: Not only physical, but also cognitive. Int. J. Geriatr. Psychiatry.

[39] Krumholz H.M. (2013). Post-hospital syndrome—An acquired, transient condition of generalized risk. N. Engl. J. Med..

[40] Schattner A. (2023). Post-discharge syndrome in older adults. Int. J. Med..

[41] Hamazaki N., Kamiya K., Yamamoto S., Nozaki K., Ichikawa T., Matsuzawa R., Yamashita M., Uchida S., Maekawa E., Meguro K. (2022). Associations between kidney function and outcomes of comprehensive cardiac rehabilitation in patients with heart failure. Clin. Res. Cardiol..

[42] Zelle D.M., Klaassen G., Bakker S.J.L., van Adrichem E., Corpeleijn E., Navis G. (2017). Physical inactivity: A risk factor and target for intervention in renal care. Nat. Rev. Nephrol..

[43] Thomas R.J., Beatty A.L., Beckie T.M., Brewer L.C., Brown T.M., Forman D.E., Franklin B.A., Keteyian S.J., Kitzman D.W., Regensteiner J.G. (2019). Home-based cardiac rehabilitation: A scientific statement from the American Association of Cardiovascular and Pulmonary Rehabilitation, the American Heart Association, and the American College of Cardiology. Circulation.

[44] Hughes J.W., Berry R., Brown T.M., Carlin B., Drwal K., Keteyian S.J., Prince D.Z., Wu W.C. (2025). Consensus statement on the virtual and remote delivery of cardiac and pulmonary rehabilitation and their components. J. Cardiopulm. Rehabil. Prev..

[45] Scherrenberg M., Falter M., Abreu A., Aktaa S., Busnatu S., Casado-Arroyo R., Dendale P., Dilaveris P., Locati E.T., Marques-Sule E. (2025). Standards for cardiac telerehabilitation. Eur. Heart J..

[46] Tamiya N., Noguchi H., Nishi A., Reich M.R., Ikegami N., Hashimoto H., Shibuya K., Kawachi I., Campbell J.C. (2011). Population ageing and wellbeing: Lessons from Japan’s long-term care insurance policy. Lancet.

[47] Yadav K., Lewis R.J. (2021). Immortal time bias in observational studies. JAMA.

[48] Hernán M.A., Robins J.M. (2016). Using big data to emulate a target trial when a randomized trial is not available. Am. J. Epidemiol..

[49] Funk M.J., Westreich D., Wiesen C., Stürmer T., Brookhart M.A., Davidian M. (2011). Doubly robust estimation of causal effects. Am. J. Epidemiol..

[50] Stuart E.A. (2010). Matching methods for causal inference: A review and a look forward. Stat. Sci..

[51] Popa L.M., Anderco P., Stoia O., Ichim C., Porr C. (2025). The kidney in the shadow of cirrhosis: A critical review of renal failure. Biomedicines.

