# Supplementary material for: Association Between Physical Therapy and Mortality in Older Patients with Heart Failure and Chronic Kidney Disease
Source: J Clin Med. 2026 Sep 11;15(18):7043. doi: 10.3390/jcm15187043 (PMC13607115; doi:10.3390/jcm15187043)
Supplement: Supplementary file 1 [file jcm-15-07043-s001.zip › jcm-4514448-supplementary.pdf]

Supplemental Material

- **Figure S1. Association between CKD Stage and All-cause Mortality in Older Patients with Heart Failure**

  ・・・page 2

- **Figure S2. Association between CKD Stage and Cardiovascular Rehospitalization in Older Patients with Heart Failure**

  ・・・page 3

- **Figure S3. Association between CKD Stage and Composite Outcome of All-Cause Mortality and Cardiovascular Rehospitalization in Older Patients with Heart Failure**

  ・・・page 4

- **Table S1. Missing data summary for baseline variables in the full study cohort (n = 6,359)**

  ・・・page 5

  Table S2. Subgroup Analyses of the Association between Post-Discharge Physical Therapy and All-Cause Mortality

  ・・・page 6

- **Table S3. Subgroup Analyses of the Association between Post-Discharge Physical Therapy and Cardiovascular-Related Rehospitalization**

  ・・・page 7

- **Table S4. Subgroup Analyses of the Association between Post-Discharge Physical Therapy and the Composite Outcome of All-Cause Mortality or Cardiovascular-Related Rehospitalization**

  ・・・page 8

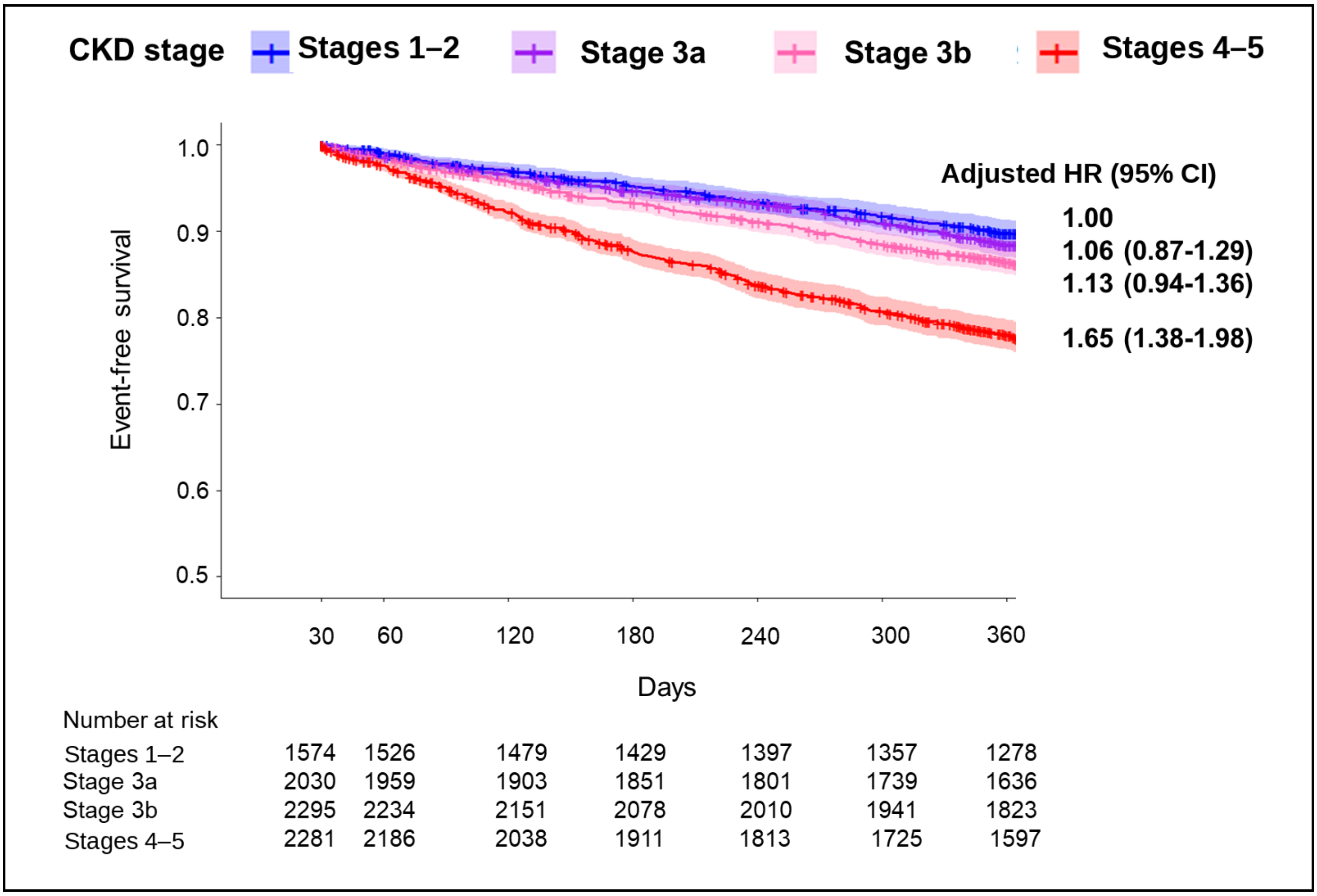

Figure S1. Association between CKD stage and all-cause mortality in older patients with heart failure (overall cohort prior to the prespecified eGFR-based exclusion).

In the prespecified 30-day landmark design, follow-up started on day 30 after discharge (time zero = day 30); therefore, numbers at risk are shown from day 30 onward. Shaded areas represent 95% CIs. Adjusted HRs and 95% CIs were obtained from Cox proportional hazards models with CKD stage as the exposure, using multiple imputation for missing covariates and pooling estimates across imputations with Rubin's rules. The models were adjusted for the same prespecified prognostic covariates as those in the primary analyses, except for CKD stage; CKD stages 1–2 served as the reference category.

CI, confidence interval; CKD, chronic kidney disease; eGFR, estimated glomerular filtration rate; HR, hazard ratio.

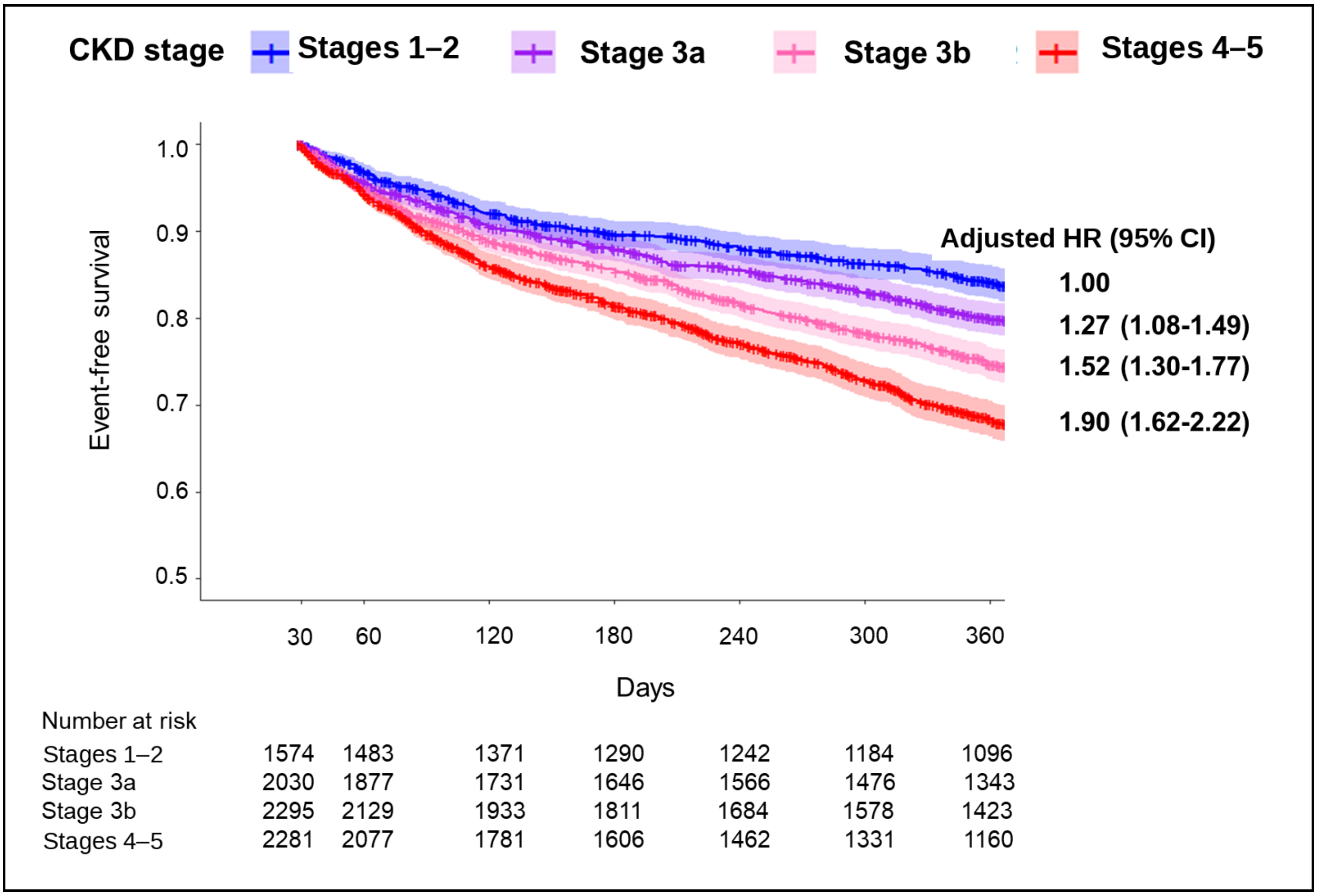

Figure S2. Association between CKD stage and cardiovascular-related rehospitalization in older patients with heart failure (overall cohort prior to the prespecified eGFR-based exclusion).

In the prespecified 30-day landmark design, follow-up started on day 30 after discharge (time zero = day 30); therefore, numbers at risk are shown from day 30 onward. Shaded areas represent 95% CIs. Adjusted HRs and 95% CIs were derived from Cox proportional hazards models with CKD stage as the exposure, using multiple imputation and Rubin's rules for pooling. Models included the same prespecified prognostic covariates as those in the primary analyses, except for CKD stage; CKD stages 1–2 served as the reference category.

CI, confidence interval; CKD, chronic kidney disease; eGFR, estimated glomerular filtration rate; HR, hazard ratio.

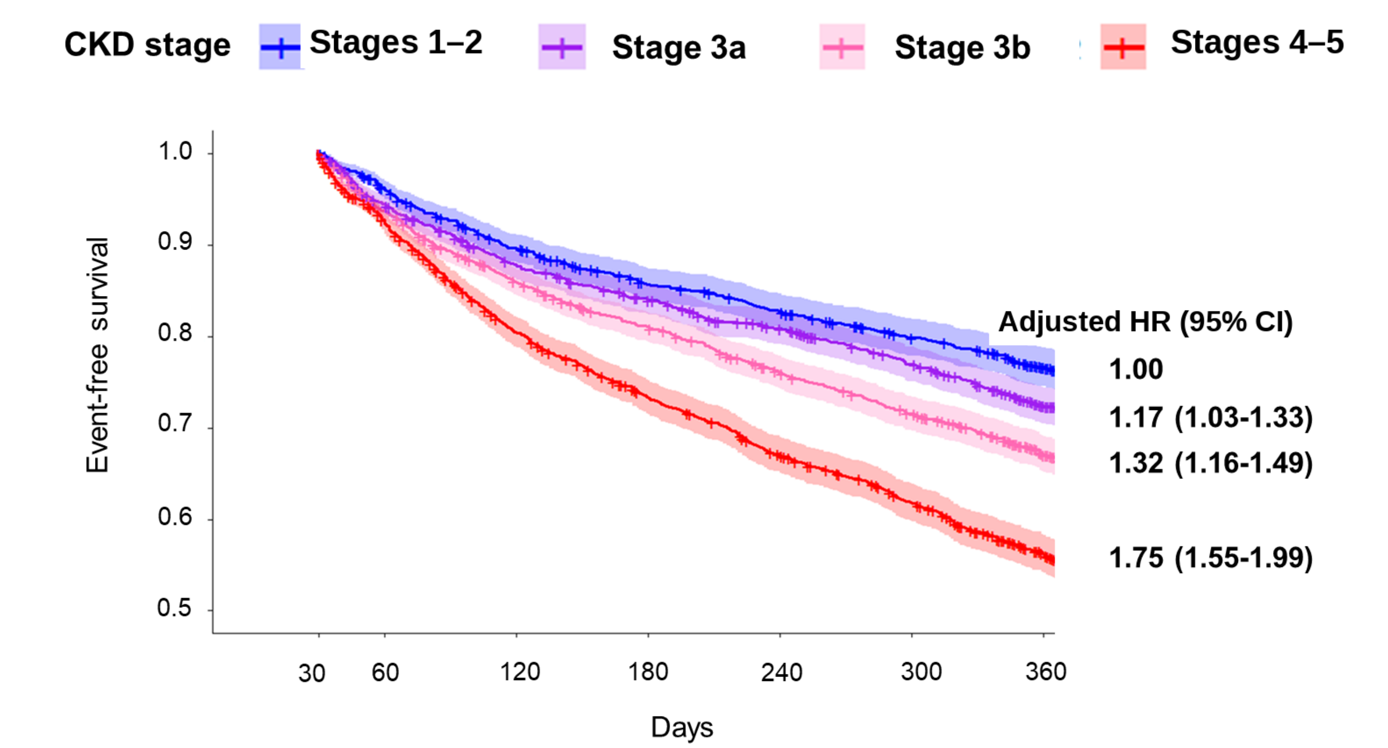

Number at risk							
Stages 1–2	1574	1483	1371	1290	1242	1184	1096
Stage 3a	2030	1877	1731	1646	1566	1476	1343
Stage 3b	2295	2127	1932	1810	1683	1577	1423
Stages 4–5	2281	2077	1781	1606	1462	1331	1160

Figure S3. Association between CKD stage and the composite outcome (all-cause mortality or cardiovascular-related rehospitalization) in older patients with heart failure (overall cohort prior to the prespecified eGFR-based exclusion).

In the prespecified 30-day landmark design, follow-up started on day 30 after discharge (time zero = day 30); therefore, numbers at risk are shown from day 30 onward. Shaded areas represent 95% CIs. Adjusted HRs and 95% CIs were obtained from Cox proportional hazards models with CKD stage as the exposure after multiple imputation, with pooling across imputations using Rubin’s rules. Models were adjusted for the same prespecified prognostic covariates as those in the primary analyses, except for CKD stage; CKD stages 1–2 served as the reference category. CI, confidence interval; CKD, chronic kidney disease; eGFR, estimated glomerular filtration rate; HR, hazard ratio.

Table S1. Missing data summary for baseline variables in the full study cohort (n = 6,359)

	Missing, n (%)
Age	0 (0)
Sex	0 (0)
Body mass index	93 (1.5)
Current smoker	7 (0.1)
NYHA functional class	11 (0.2)
History of heart failure	0 (0)
Chronic kidney disease stage	0 (0)
Comorbidities	
Hypertension	0 (0)
Diabetes mellitus	0 (0)
Dyslipidemia	0 (0)
Chronic obstructive pulmonary disease	0 (0)
Cerebrovascular disease	0 (0)
Cancer	0 (0)
Musculoskeletal disorders	0 (0)
Dementia	3 (0.1)
Peripheral arterial disease	0 (0)
Etiology of heart failure	
Ischemic heart disease	0 (0)
Valvular heart disease	0 (0)
Cardiomyopathy	0 (0)
Arrhythmia	0 (0)
Echocardiography	
LVEF	0 (0)
Left atrial diameter	348 (5.5)
Blood biochemistry tests	
BNP	130 (2.0)
Serum creatinine	0 (0)
eGFR	0 (0)
BUN	0 (0)
Serum albumin	167 (2.6)
Hemoglobin	11 (0.2)
Sodium	8 (0.1)
Medication use	

ACE inhibitor/ARB/ARNI	5 (0.1)
Mineralocorticoid receptor antagonist	0 (0)
β-blocker	5 (0.1)
SGLT2 inhibitor	7 (0.1)
Diuretic	5 (0.1)
Kihon Checklist score	742 (11.7)
Living alone	0 (0)
Barthel Index at discharge	151 (2.4)
Grip strength	888 (14.0)
Comfortable walking speed	1143 (18.0)

Missing data were assessed across all baseline variables used in multiple imputation and propensity score estimation. Variables with no missing values are denoted as 0 (0). Multiple imputation by chained equations (MICE) was performed with 50 imputed datasets to handle missing values prior to propensity score matching. Variables with notably high rates of missingness included comfortable walking speed (18.0%), grip strength (14.0%), Kihon Checklist score (11.7%), and left atrial diameter (5.5%). These variables were retained in the imputation model given their clinical relevance as prognostic indicators in older patients with heart failure.

ACE, angiotensin-converting enzyme; ARB, angiotensin receptor blocker; ARNI, angiotensin receptor–neprilysin inhibitor; BNP, B-type natriuretic peptide; BUN, blood urea nitrogen; eGFR, estimated glomerular filtration rate; LVEF, left ventricular ejection fraction; NYHA, New York Heart Association; SGLT2, sodium–glucose cotransporter 2.

Table S2. Subgroup Analyses of the Association between Post-Discharge Physical Therapy and All-Cause Mortality

Subgroup	Category	HR	95% CI	P value	P for interaction
CKD stage	3a	NE*	0.00–Inf	0.998	0.062
	3b	0.80	0.24–2.62	0.710	
	4–5	0.55	0.18–1.64	0.282	
Age	<80 y	0.46	0.16–1.32	0.148	0.981
	≥80 y	0.45	0.15–1.31	0.141	
Sex	Female	0.46	0.08–2.49	0.364	0.658
	Male	0.41	0.17–0.99	0.047	
HFrEF	No	1.16	0.31–4.31	0.827	0.096
	Yes	0.29	0.11–0.79	0.015	
Ischemic heart disease	No	0.56	0.22–1.42	0.221	0.507
	Yes	0.32	0.09–1.20	0.092	
HF history	None	0.46	0.17–1.22	0.118	0.957
	>1 year before	0.21	0.03–1.78	0.153	
	≤1 year before	0.49	0.08–2.91	0.430	
HT	No	0.32	0.10–1.02	0.053	0.441
	Yes	0.57	0.21–1.55	0.274	
DM	No	0.32	0.10–1.01	0.052	0.586
	Yes	0.53	0.18–1.56	0.252	
Frailty	No	0.46	0.22–0.97	0.042	>0.99
	Yes	0.38	0.16–0.90	0.028	
ACEI/ARB/ARNI use	No	1.27	0.21–7.64	0.791	0.233
	Yes	0.38	0.16–0.91	0.030	
Beta-blocker use	No	0.32	0.03–3.08	0.324	0.722
	Yes	0.48	0.22–1.08	0.075	
SGLT2 use	No	0.55	0.21–1.38	0.202	0.571
	Yes	0.35	0.09–1.29	0.114	

*Not estimable because no deaths occurred in the PT group.

HRs and 95% CIs were derived from Cox proportional hazards models.

ACEI, angiotensin-converting enzyme inhibitor; ARB, angiotensin II receptor blocker; ARNI, angiotensin receptor–neprilysin inhibitor; CI, confidence interval; CKD, chronic kidney disease; DM, diabetes mellitus; HF, heart failure; HFrEF, heart failure with reduced ejection fraction; HR, hazard ratio; HT, hypertension; SGLT2, sodium–glucose cotransporter 2.

Table S3. Subgroup Analyses of the Association between Post-Discharge Physical Therapy and Cardiovascular-Related Rehospitalization

Subgroup	Category	HR	95% CI	P value	P for interaction
CKD stage	3a	0.82	0.42–1.64	0.580	0.950
	3b	0.83	0.46–1.50	0.538	
	4–5	0.73	0.40–1.31	0.290	
Age	<80 y	0.72	0.45–1.15	0.169	0.623
	≥80 y	0.88	0.50–1.55	0.663	
Sex	Female	0.71	0.39–1.29	0.261	0.677
	Male	0.83	0.53–1.30	0.415	
HFrEF	No	0.93	0.52–1.66	0.803	0.483
	Yes	0.71	0.45–1.12	0.137	
Ischemic heart disease	No	0.81	0.53–1.24	0.335	0.747
	Yes	0.71	0.37–1.37	0.309	
HF history	None	0.54	0.32–0.92	0.023	0.075
	>1 year before	1.03	0.57–1.89	0.914	
	≤1 year before	1.59	0.64–3.93	0.319	
HT	No	0.81	0.40–1.62	0.547	0.980
	Yes	0.80	0.53–1.21	0.293	
DM	No	0.66	0.39–1.12	0.125	0.339
	Yes	0.94	0.58–1.53	0.792	
Frailty	No	0.78	0.55–1.12	0.178	>0.99
	Yes	0.70	0.43–1.15	0.162	
ACEI/ARB/ARNI use	No	1.02	0.52–2.00	0.960	0.375
	Yes	0.71	0.46–1.09	0.115	
Beta-blocker use	No	0.64	0.29–1.37	0.247	0.527
	Yes	0.83	0.56–1.24	0.368	
SGLT2 use	No	0.74	0.48–1.16	0.187	0.644
	Yes	0.88	0.48–1.62	0.691	

HRs and 95% CIs were derived from the Cox proportional hazards models.

ACEI, angiotensin-converting enzyme inhibitor; ARB, angiotensin II receptor blocker; ARNI, angiotensin receptor–neprilysin inhibitor; CI, confidence interval; CKD, chronic kidney disease; DM, diabetes mellitus; HF, heart failure; HFrEF, heart failure with reduced ejection fraction; HR, hazard ratio; HT, hypertension; SGLT2, sodium–glucose cotransporter 2.

Table S4. Subgroup Analyses of the Association between Post-Discharge Physical Therapy and the Composite Outcome of All-Cause Mortality or Cardiovascular-Related Rehospitalization

Subgroup	Category	HR	95% CI	P value	P for interaction
CKD stage	3a	0.61	0.32–1.17	0.137	0.836
	3b	0.78	0.44–1.35	0.372	
	4–5	0.64	0.36–1.12	0.119	
Age	<80 y	0.64	0.41–1.00	0.048	0.758
	≥80 y	0.72	0.43–1.20	0.205	
Sex	Female	0.60	0.34–1.08	0.089	0.640
	Male	0.72	0.47–1.08	0.113	
HFrEF	No	0.83	0.48–1.43	0.494	0.385
	Yes	0.60	0.39–0.92	0.019	
Ischemic heart disease	No	0.70	0.47–1.04	0.080	0.728
	Yes	0.61	0.33–1.13	0.117	
HF history	None	0.48	0.29–0.78	0.003	0.079
	>1 year before	0.92	0.51–1.65	0.781	
	≤1 year before	1.24	0.53–2.85	0.621	
HT	No	0.59	0.32–1.11	0.104	0.563
	Yes	0.73	0.49–1.08	0.114	
DM	No	0.57	0.35–0.93	0.024	0.314
	Yes	0.81	0.51–1.28	0.360	
Frailty	No	0.67	0.48–0.94	0.021	>0.99
	Yes	0.59	0.37–0.94	0.026	
ACEI/ARB/ARNI use	No	0.95	0.50–1.81	0.866	0.233
	Yes	0.60	0.40–0.89	0.012	
Beta-blocker use	No	0.53	0.25–1.12	0.096	0.463
	Yes	0.72	0.49–1.05	0.085	
SGLT2 use	No	0.66	0.44–1.01	0.053	0.831
	Yes	0.71	0.40–1.25	0.240	

HRs and 95% CIs were derived from Cox proportional hazards models.

ACEI, angiotensin-converting enzyme inhibitor; ARB, angiotensin II receptor blocker; ARNI, angiotensin receptor–neprilysin inhibitor; CI, confidence interval; CKD, chronic kidney disease; DM, diabetes mellitus; HF, heart failure; HFrEF, heart failure with reduced ejection fraction; HR, hazard ratio; HT, hypertension; SGLT2, sodium–glucose cotransporter 2.
